# A Simple Solution of the Mystery of Tubercles

**Published:** 1883-04

**Authors:** 


A Simple Solution of the Mystery of Tubercles, etc. By R. R. Gregg, M. D.

This pamphlet of Dr. Gregg's has been very interesting reading to the writer. Whether or not it *solves* the question of tubercles we cannot say, not being a pathological expert. The question is certainly a knotty one and sadly needs some one to solve it.

